# Effects of host species on microbiota composition in *Phlebotomus* and *Lutzomyia* sand flies

**DOI:** 10.1186/s13071-023-05939-2

**Published:** 2023-08-31

**Authors:** Ahmed Tabbabi, Daiki Mizushima, Daisuke S. Yamamoto, Hirotomo Kato

**Affiliations:** Division of Medical Zoology, Department of Infection and Immunity, Jichi Medical University, Shimotsuke, Tochigi 329-0498 Japan

**Keywords:** Sand flies, *Phlebotomus*, *Lutzomyia*, Microbiota, *Leishmania*, Host phylogeny

## Abstract

**Background:**

Blood-sucking phlebotomine sand flies are vectors of the protozoan parasites *Leishmania* spp. Although the intestinal microbiota is involved in a wide range of biological and physiological processes and has the potential to alter vector competence, little is known about the factors that modify the gut microbiota composition of sand flies. As a key step toward addressing this issue, we investigated the impact of host species on the gut bacterial composition in *Phlebotomus* and *Lutzomyia* sand flies reared under the same conditions.

**Methods:**

Bacterial 16S rRNA gene amplification and Illumina MiSeq sequencing were used to characterize the overall bacterial composition of three laboratory-reared sandflies: *Phlebotomus papatasi*, *Ph. duboscqi*, and *Lutzomyia longipalpis.*

**Results:**

Our results showed that the larvae of the three sand fly species harbored almost the same microbes but had different relative abundances. Adult *Ph. papatasi* and *Ph. duboscqi* revealed similar microbiome compositions, which were distinct from that of adult *Lu. longipalpis*. Furthermore, we showed that *Ph. papatasi* and *Ph. duboscqi* are hosts for different bacterial genera. The experiment was repeated twice to improve accuracy and increase reliability of the data, and the same results were obtained even when a distinct composition of the microbiome among the same species was identified probably because of the use of different larvae food batch.

**Conclusions:**

The present study provides key insights into the role of host species in the gut microbial content of different sand fly species reared under the same conditions, which may influence their susceptibility to *Leishmania* infection.

**Graphical Abstract:**

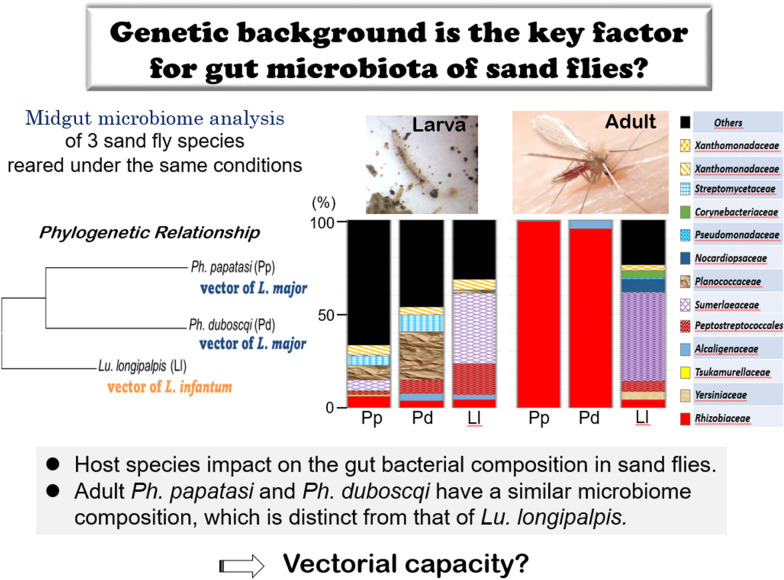

**Supplementary Information:**

The online version contains supplementary material available at 10.1186/s13071-023-05939-2.

## Background

Phlebotomine sand flies (Diptera: Psychodidae) are hematophagous insects that feed on a wide range of hosts and transmit a vast array of pathogens responsible for causing diseases in humans and animals worldwide. Among the more than 1000 sand fly species that have been validated to date, only 10% are known or suspected vectors of different pathogens, including arboviruses and bacteria, but they are well recognized as the main vectors of *Leishmania*, the causative agent of leishmaniasis, a neglected tropical disease [1, 2].

Sand flies live in groups and interact with diverse microbiota. It has been demonstrated that microbial symbionts influence key aspects of their insect host’s fitness [3–6]. According to a previous study, *Lutzomyia* *longipalpis* flies fed a diet containing rabbit feces were more likely to lay eggs than flies fed sterilized feces of the larvae stage [3]. Additionally, delayed hatching and lower survival rates were observed in larvae fed sterile feces. The reintroduction of eliminated bacteria confirmed the initial findings, suggesting the importance of bacterial presence and specificity for sand fly development. Bacteria belonging to the phylum *Proteobacteria* participate in insect host nutrition by fixing atmospheric nitrogen [7]. Conversely, the host can also control the microbial composition to some extent, such as by changing nutrient availability through diet choice, host metabolism [8], or by triggering immune factors [9]. It has been demonstrated in this context that sand flies from tropical regions including *Lu. longipalpis* seemingly bred in soil enriched with decomposed leaves and other detritus, with a preference for tree bases. Additionally, insects tend to mount immune responses to maintain a complex balance between acceptance and rejection, thereby maintaining a peaceful coexistence [9].

The natural gut microbiota is acquired by adult sand flies from various sources, including sugarcane plants and blood from a wide range of hosts or from re-colonization of the gut by microbes ingested by terrestrial-dwelling larval stages [3, 10–13]. Most larval-stage bacteria undergo biodegradation during the pupal stage, and the microbial charge is immediately and significantly reduced after the emergence of the adult [14, 15]. Female sand flies become infected by ingesting infected cells during blood meals and create an interactive relationship between the microbial community of the gut and the parasite, because the developmental life cycle of *Leishmania* within the sand fly vector occurs exclusively in the mid- and hindgut in the presence of symbiotic bacteria [16, 17]. The gut bacterial community in sand flies may exert a negative or positive effect on the development of *Leishmania* depending on the bacterial species [14, 17–21]. Further studies on the link between bacteria deposited during *Leishmania*-infected sand fly bites and the clinical outcomes of leishmaniasis have suggested that the gut microbes of *Lu. longipalpis* are egested into the host skin besides *Leishmania* triggering neutrophil infiltration and facilitating parasitic installation [22]. According to previous studies, particular attention has been given to the interactions occurring between the microbial community of the sand fly gut and parasites, and a pool of symbiotic microbiota has been considered as a potential candidate for paratransgenic or biological approaches for the control of sand fly populations [9, 11]. Previous studies have shown that variations in the insect gut microbiota may be expressed by many factors, including host habitat, diet, developmental stage, and phylogeny, all of which contribute to the composition of the insect gut microbiota [23–28]. However, little is known about the host genetic factors that modify the gut microbiota composition of sand flies, and to the best of our knowledge, no research has been conducted on these insects under controlled conditions.

Several species of *Phlebotomus* and *Lutzomyia* sand flies, which are geographically distributed across the tropics, have been identified as major vectors of leishmanial parasites [29]. Each sand fly species occupies a specific ecological niche and has a climate-sensitive life cycle, and its population biology is governed by a mixture of abiotic and biotic factors that act independently and through interactions. The sand flies *Phlebotomus* (*Ph.*) *papatasi* and *Phlebotomus* (*Ph.*) *duboscqi* are Old World vectors of *Leishmania* (*L.*) *major*, the etiological agent of cutaneous leishmaniasis. In contrast, *Lu. longipalpis* sand flies are the major natural vectors of *Leishmania* (*L.*) *infantum* parasites responsible for the transmission of visceral leishmaniasis in the New World. These three phlebotomine sand flies are widely colonized in different international laboratories and are used as live vector models in a diverse array of research projects.

The present study aimed to test whether host species affect the composition of the gut microbiota in the three sand fly species cited above reared under the same conditions, minimizing the differences brought about by diet and environmental factors. Because the features of each species differ within *Phlebotomus* and *Lutzomyia* sand flies, we predicted that there would also be alterations in their microbiomes.

## Methods

### Origin and maintenance of sand flies

Three laboratory-reared sand fly species were used: two Old World species [*Phlebotomus papatasi* from Jordan (PPJO) and *Ph. duboscqi* from Mali (PDMA)] and one New World species [*Lu. longipalpis* from Jacobina (LLJB)]. These strains were obtained from the Vector Molecular Biology Section, Laboratory of Malaria and Vectors, USA, and maintained in the Division of Medical Zoology at Jichi Medical University, Japan. They were reared for > 5 years under the same laboratory conditions to minimize the potential influence of environmental factors and diet. In the laboratory, the sand flies were maintained in net cages (30 × 30 × 30 cm) at 26 ± 1 °C, 80–90% humidity, and a 12:12 (light: dark) photoperiod. The sand flies had access to a piece of cotton soaked in 30% sucrose solution. Non-blood-fed females were exposed to BALB/c mice (Japan SLC, Shizuoka, Japan) as the blood source. The blood-fed females were transferred individually to vials for oviposition. Eggs were placed in a 150-ml polystyrene container filled with 2 cm of plaster of Paris at its bottom (oviposition container) and were kept in the dark at 26 ± 1 °C, 80–90% humidity. Just before hatching, a very small amount of food was sprinkled on several spots near the eggs. Larval food was prepared by mixing fresh rabbit feces and rabbit chow in a 1:1 ratio, which was fermented at 26 °C for 4 weeks in a dark chamber, air dried, and ground to a powder [30]. Notably, two different food batches were prepared separately at two time different intervals, which may have caused change in bacterial communities.

### Experimental infections of sand flies

Female sand flies were experimentally infected by feeding through a chick-skin membrane on heat-inactivated blood containing 10^6^ *L. major* promastigotes per milliliter. The parasite viability was checked after blood feeding. Engorged sand flies were then separated out and kept at 26 °C under standard conditions [30]. The promastigotes are seen upon microscopic examination of gut specimens at 3 days’ post blood meal.

### Sample preparation and bacterial DNA extraction

Bacterial DNA was extracted from all eight larvae at stage L4, and a pool of eight dissected midguts of adults from the three laboratory-reared species of sand flies at different time intervals: 1 week after release, before feeding, and 3 and 7 days post-feeding. The same sample collection protocol was followed to extract DNA from infected and uninfected blood-fed sand flies at 3 days post-feeding. Whole sand fly samples were first washed with distilled water, followed by 70% ethanol, and rinsed thrice with sterile phosphate buffered saline (PBS) and finally with double-distilled water. After washing, sand fly guts were gently dissected under a stereomicroscope on sterilized single-use slide covers using sterile insect needles.

Pooled samples were homogenized in 0.5 ml Micrewtube® containing 250 µl of phosphate-buffered saline (PBS) or saline. Approximately 0.5 g of 0.1 mm-diameter zirconia/silica beads (BioSpec Products, Bartlesville, OK, USA) were added to the extraction tubes to mechanically crush microbial cells using a ShakeMan6 bead crusher (Bio Medical Science, Tokyo, Japan) [31, 32]. Crushed cells were spun down at high speeds to obtain a precipitate of cell debris, and 200 µl of the supernatant was subjected to DNA isolation. Genomic DNA was purified from sand fly pools using the ReliaPrep DNA Cleanup and Concentration System kit (Promega Corporation, Madison, WI, USA), according to the manufacturer’s instructions. The same protocol was followed to extract bacterial DNA from the larval food.

### 16S RNA gene-based identification of bacteria from sand flies by PCR and Illumina MiSeq Sequencing

Two PCR steps were performed to amplify the variable region (V3–V4) of the 16S rRNA gene. In the first step, PCR amplification was performed in a thermocycler after DNA extraction using primers targeting the V3 and V4 regions of the 16S rRNA gene. The following primers were used to amplify the hypervariable regions V3-V4 of the 16S rRNA gene: forward primer: (5ʹTCGTCGGCAGCGTCAGATGTGT ATAAGAGACAGCCTACGGGNGGCWGCAG-3ʹ) and reverse primer (5ʹGTCTCGT GGGCTCGGAGATGTGTATAAGAGACAGGACTACHVGGGTATCTAATCC-3ʹ). These regions were approved by the Illumina protocol manual [33] and yielded high-quality sequence data as described previously [34]. PCR amplification was executed with 35 cycles of denaturation (95 °C, 30 s), annealing (55 °C, 30 s), and polymerization (72 °C, 30 s) using AmpliTaq Gold 360 DNA polymerase. Index primers were used to reamplify each portion of the PCR product and generate amplicon libraries for Illumina sequencing [33]. The amplified products were subjected to electrophoresis on a 1.5% agarose gel. Sequencing was performed using the Illumina MiSeq platform with MiSeq Reagent Kit version 3 (Illumina Inc., San Diego, CA, USA).

### Quantification of gut bacteria in sand flies

The gut bacteria in sand flies were quantified by qPCR with TB Green Fast Mix (TOYOBO Co., Ltd., Osaka, Japan) using a Thermal Cycler Dice Real Time System Lite (Takara Bio Inc., Shiga, Japan). The bacterial 16S rRNA gene was used as the target, and glyceraldehyde 3-phosphate dehydrogenase (*GAPDH*) was used as the reference. The primer sequences were 5ʹ-ACHCCTACGGGDGGCWGCAG-3ʹ (16S-q-337F) and 5ʹ-GTDTYACCGCGGYTGCTGGCAC-3ʹ (16S-q-514R) for the amplification of bacterial 16S rRNA gene, and 5ʹ-TTCGCAGAAGACAGTGATGG-3ʹ (Lugapdh-q-F) and 5ʹ-CCCTTCATCGGTCTGGACTA-3ʹ (Lugapdh-q-R), and 5ʹ-CGACTTCAACAGCA ACTCCCACTCTTCC-3ʹ (Phgapdh-q-F) and 5ʹ-TGGGTGGTCCAGGGTTTCTTACT CCTT-3ʹ (Phgapdh-q-R) for gapdh gene [35, 36]. Relative quantities of 16S rRNA genes were determined by ∆∆Ct method using *GAPDH* as the reference.

### ITS1-based identification of fungus from sand flies by PCR and Illumina MiSeq Sequencing

The fungal ITS1 region was independently amplified using two PCR steps as described above for bacterial DNA. In view of the different kinds of biases (specificity to fungi, mismatches, length, and taxonomy), different primer combinations targeting different parts of the ITS region were used simultaneously, as suggested by the Illumina protocol manual [37]. The ITS regions were sequenced using the Illumina MiSeq platform and MiSeq reagent kit version 3 (Illumina).

### Data processing and analysis

The reads generated from the MiSeq platform (600 cycles, paired-end format) were exported as FASTQ files for importing into Quantitative Insights Into Microbial Ecology 2 (QIIME2) (version 2020.2.0) [38]. The paired-end reads were trimmed and merged using the DADA2 program in the QIIME2 Plugin. Sequences were clustered in amplicon sequences variants (ASVs) by the QIIME2 program. The OTUs were annotated based on the SILVA version 138 dataset [39] with 99% sequence identity.

Principle coordinate analysis (PCoA) on Bray-Curtis dissimilarity was used to display the beta diversity indices of bacterial communities among sand flies [40]. The group significance beta diversity indexes were calculated with QIIME2 plugins using a permutational multivariate analysis of variance (PERMANOVA), respectively. A *P*-value < 0.05 was considered significant.

## Results

### Microbiome profile of laboratory-reared sand flies

In this study, we characterized the microbiota content of three sand fly species reared under the same conditions, minimizing differences caused by diet and environmental factors, to test whether host species affect bacterial diversity. To characterize the bacterial lineages present in the midgut microbiota, we performed multiplex Illumina sequencing of the V3 and V4 hypervariable regions of the 16S rRNA gene. The experiment was repeated twice (experiment 1 and 2) to obtain accurate and reliable data, and family level analysis was used when the identification of bacterial genera failed. The most dominant sequences in the two repeated experiments belonged to two bacterial families, Rhizobiaceae and Yersiniaceae, belonging to *Proteobacteria* phylum.

**Fig. 1:**
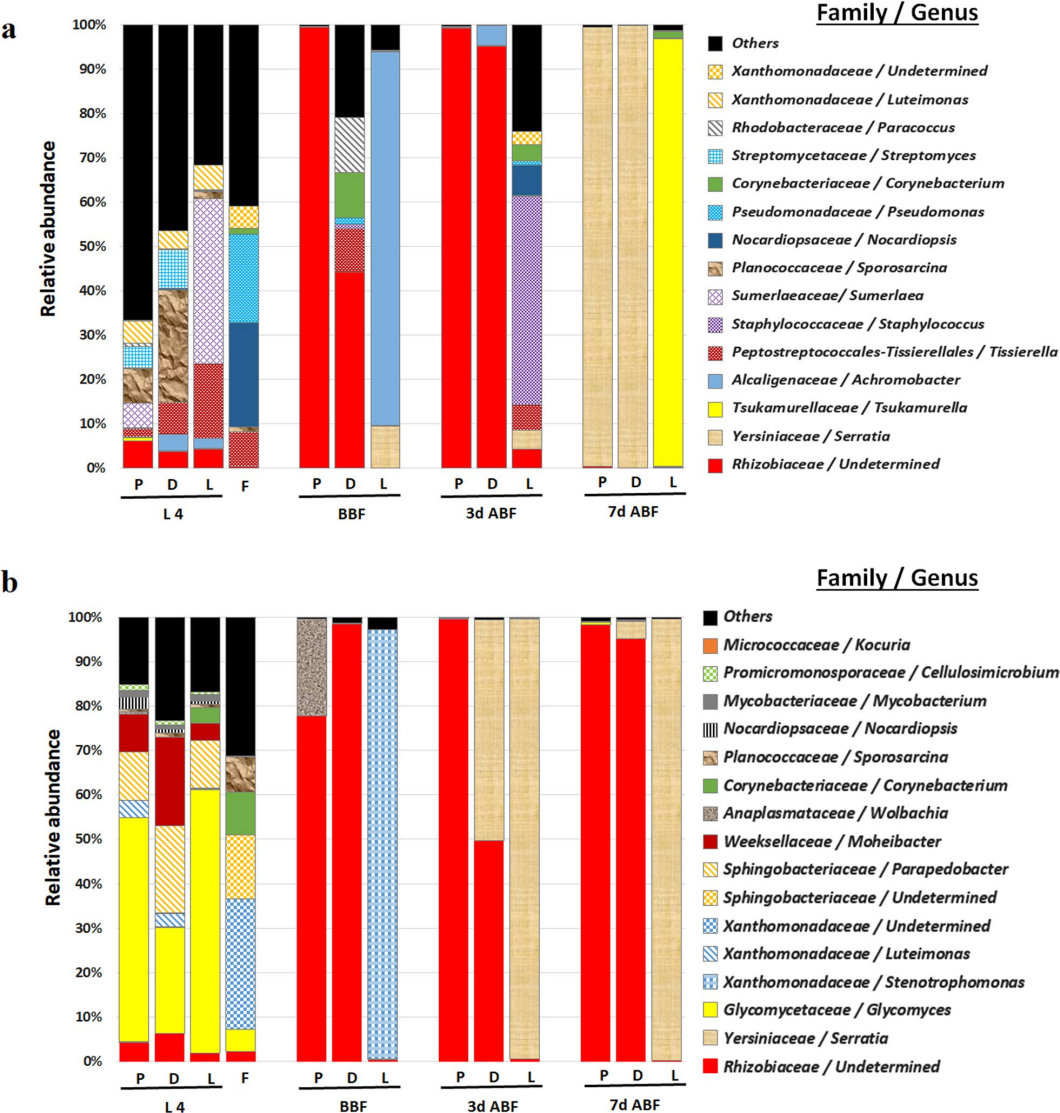
**a** Microbiota taxa at family and genus levels of three laboratory-reared sand flies in experiment 1. **b** Microbiota taxa at family and genus levels of three laboratory-reared sand flies in experiment 2. P, *Phlebotomus papatasi*; D, *Ph. duboscqi*; L, *Lutzomyia longipalpis*; L4, larval stage 4; F, food; BBF, before blood feeding; 3d ABF, 3 days after blood feeding; 7d ABF, 7 days after blood feeding

The taxa of the microbiota at the family and genus levels are shown in Fig. 1a (Experiment 1). The larvae of *Phlebotomus* and *Lutzomyia* species harbored similar microbes, which were represented by different relative abundances. Based on the relative abundance data and beta diversities analysis, *Ph. papatasi* and *Ph. duboscqi* were close to each other and distant from *Lu. longipalpis* (Additional file 1: Fig. S1a; PERMANOVA, *Df* = 2, *P* = 0.347). *Lutzomyia longipalpis* contains more *Sumerlaea* and less *Sporosarcina* than *Ph. papatasi* and *Ph. duboscqi*, which belong to Sumerlaeaceae and Planococcaceae, respectively (Fig. 1a). Moreover, two main bacterial genera were identified in used larval food, including *Pseudomonas* and *Nocardiopsis* (belonging to *Pseudomonadaceae* and *Nocardiopsaceaea* families, respectively), which were present only in some sand fly microbiota. Additionally, bacteria with low percentages grew slightly in some larval and adult sand flies (*Tissierella*, *Sporosarcina*, *Corynebacterium*, Xanthomonadaceae). The gut bacterial communities of adult *Ph. papatasi* and *Ph. duboscqi* were similar at 3 and 7 days post-feeding and were dominated by Rhizobiaceae (99.3% for *Ph. papatasi* and 95.3% for *Ph. duboscqi*) and *Serratia* belonging to Yersiniaceae family (99.1% for *Ph. papatasi* and 99.9% for *Ph. duboscqi*) (Fig. 1a), respectively. The Rhizobiaceae family was shared across the two freshly released *Ph. papatasi* (99.4%) and *Ph. duboscqi* (44.2%) 7 days after release and before feeding, whereas *Ph. duboscqi* comprised individuals of particular genera [*Tissierella* (9.8%), *Corynebacterium* (10.1%), and *Paracoccus* (12.4%) belonging to the Tissierellaceae, Corynebacteriaceae, and Rhodobacteraceae families, respectively]. In this study, the microbial communities in species of the genus *Phlebotomus* were close to each other, whereas those in *Lu. longipalpis* were distant from each other. Three main bacterial genera were identified in the gut of the last sand fly species, freshly released, and 3 and 7 days post-feeding, including *Achromobacter* (84.3%), *Staphylococcus* (46.9%), and *Tsukamurella* (96.2%), respectively [belonging to Alcaligenaceae, Staphylococcaceae, and Tsukamurellaceae families, respectively].

The gut microbiota was quantified by qPCR to determine the changes in bacterial communities in different sand flies (Experiment 1). Our results showed that the microbial charge markedly reduced after the emergence of the adults in *Ph. papatasi* (≃ six times). However, the bacterial number increased significantly after feeding (on blood) in *Ph. papatasi* and *Ph. duboscqi* (≃ three and two times, respectively), and almost the same bacterial charge was retained 7 days after blood feeding (Additional file 2: Fig. S2a).

The taxa of the bacterial communities at the family and genus levels are shown in Fig. 1b (Experiment 2). Based on the diversity data, bacterial communities were very similar among the three larval sand fly species. As recorded in experiment 1, adult *Ph. papatasi* and *Ph. duboscqi* were close to each other and distant from adult *Lu. Longipalpis* (Additional file 1: Fig. S1b; PERMANOVA, *Df* = 2, *P* = 0.004). Compared to the first experiment, a complete change in bacterial composition was observed in the larval foods of the two batches linked probably because of bacterial contamination during processing and handling. Xanthomonadaceae and Sphingobacteriaceae families were the main bacteria identified in the food, but were not present in the sand fly microbiota. However, low percentages of bacteria grew in some larvae and adult sand flies (*Glycomyces*, Rhizobiaceae). In the adults, Rhizobiaceae and Yersiniaceae (*Serratia*) were the dominant microbial families in experiment 2. Rhizobiaceae were highly abundant in all adult *Ph. papatasi* and *Ph. duboscqi* and completely absent in *Lu. longipalpis*, in which Yersiniaceae (99.3% and 99.8% at 3 and 7 days post-feeding, respectively) and *Stenotrophomonas* belonging to Xanthomonadaceae (96.7% in sand flies before feeding) constituted almost all the microbiota communities.

In experiment 2, the microbial charge significantly reduced after the emergence of the adults in *Ph. papatasi* (≃ 6 times). However, the bacterial number increased (≃2, 18, and 7 times in *Ph. papatasi, Ph. duboscqi*, and *Lu. longipalpis*, respectively) 3 days after blood feeding and decreased again to reach a very low bacterial quantity on day 7 post-feeding (Additional file 2: Fig. S2b).

### Bacterial communities in uninfected and infected sand flies

**Fig. 2:**
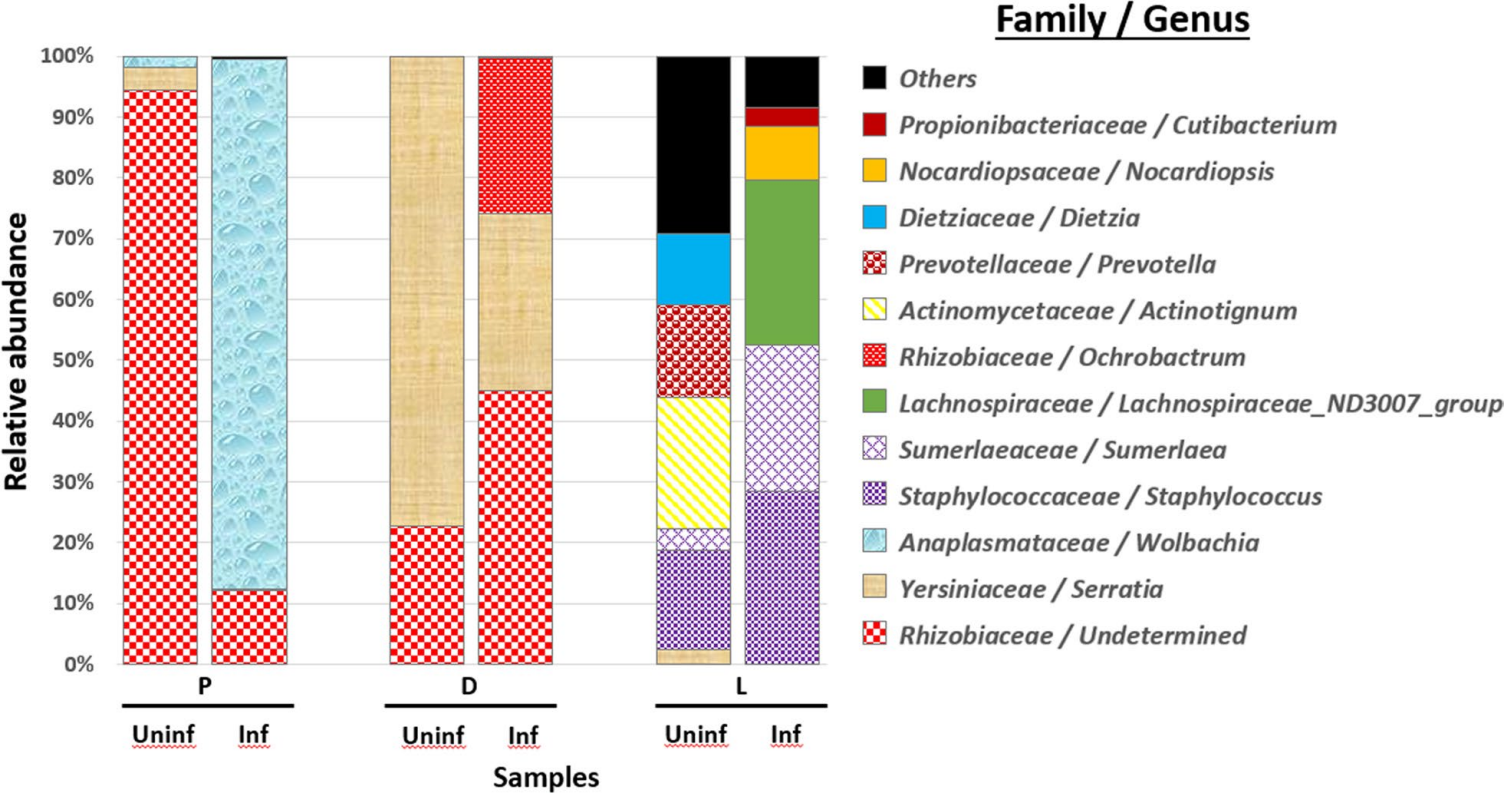
Bacterial composition of uninfected and infected laboratory-reared sand flies in experiment 1. P, *Phlebotomus papatasi*; D, *Ph. duboscqi*; L, *Lutzomyia longipalpis*; Uninf, uninfected; Inf, infected

The diversity of the sand fly gut microbiome was compared between the control and infected sand flies 3 days after blood feeding (Fig. 2). As stated above, a fraction of the bacterial genera and families were shared by the two closely related *Phlebotomus* species, whereas a special composition was observed in *Lu. longipalpis*. In addition, we found that the diversity and abundance of the gut bacterial communities between *Leishmania*-infected and uninfected sand flies were quite different in *Phlebotomus* and *Lutzomyia* genera, respectively (Fig. 2).

Most bacteria present in the gut of the *Ph. papatasi* host belonged to Rhizobiaceae, which decreased from 94.3% in the control group to 12.2% in the infected group. However, Anaplasmataceae increased dramatically from 1.6% in uninfected individuals to 86.8% in infected individuals. Three bacterial genera belonging to two families (Fig. 2) were identified in *Ph. duboscqi*. The relative abundance of Rhizobiaceae (undetermined and *Ochrobactrum* genera) increased from 22.8% in uninfected individuals to 70.6% in infected individuals, while that of Yersiniaceae decreased from 77.1% to 29.12%. In contrast, the bacterial composition of *Lu. longipalpis* changed completely between the control and infected groups, except for *Staphylococcus* genus (Staphylococcaceae), which maintained almost the same abundance (14.2% and 25.7% in the uninfected and infected groups, respectively) (Fig. 2).

Because the gut microbiota may affect parasite development, quantitative changes in the bacterial communities were assessed. The gut microbiota was quantified by qPCR for uninfected and infected sand flies at separate times. Our results showed that the bacterial load could change slightly or markedly between both sand fly groups, suggesting a quantitative interaction with *Leishmania* parasites (Additional file 3: Fig. S3).

### Fungal profile of laboratory-reared sand flies

**Fig. 3:**
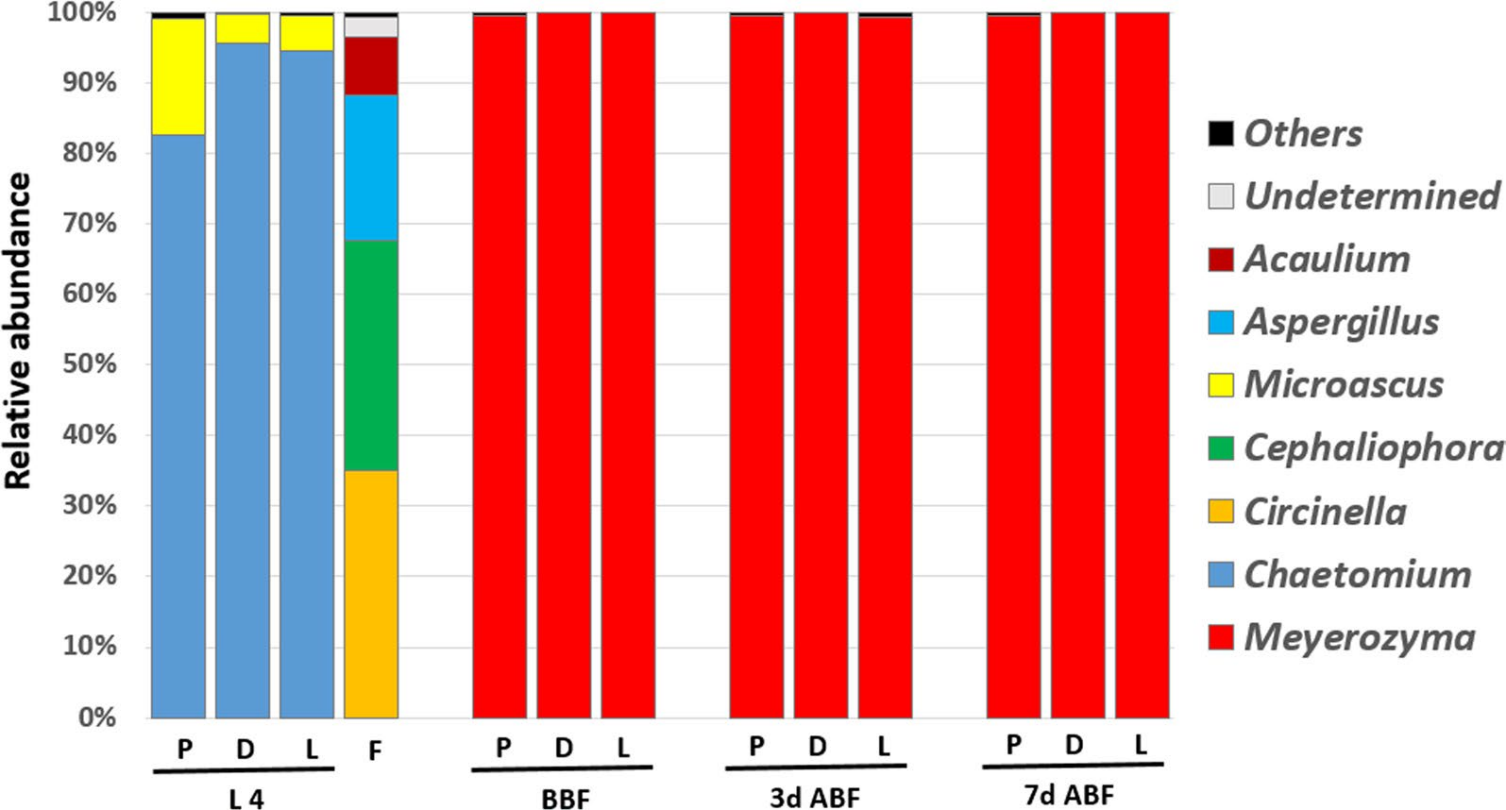
Fungal composition of three laboratory-reared sand flies. P, *Phlebotomus papatasi*; D, *Ph. duboscqi*; L, *Lutzomyia longipalpis*; L4, larval stage 4; F, food; BBF, before blood feeding; 3d ABF, 3 days after blood feeding; 7d ABF, 7 days after blood feeding

As shown in Fig. 3, *Chaetomium* and *Microascus* were the main fungal genera in the larvae of sand fly species. The fungal composition of the larval food was dominated by *Circinella* (32.7%), *Cephaliophora* (30.2%), and *Aspergillus* (19.4%) but not sand fly fungi. Additionally, *Meyerozyma* genus is the only fungus found in all adult sand flies, regardless of the species. However, the fungal composition of the control and infected sand flies was checked, and no difference was found. *Meyerozyma* was the only genus identified in both groups.

## Discussion

Microbiota communities were determined in *Phlebotomus* and *Lutzomyia* sand flies reared under the same conditions, and the effects of host species on bacterial structure were explored in the present study. Previous studies on beetles and mosquitoes revealed that microbiomes vary depending on the diet or sampling location of their hosts [26, 41]. The sand flies used in this study had lived for > 5 years under the same conditions, and it was therefore thought that other variables, such as diet, temperature, and humidity, would not strongly affect the microbiome. Notably, females of *Ph. papatasi* and *Ph. duboscqi* are Old World species that are morphologically and genetically closely related, and both are known to be vectors of *L. major* [42]. In contrast, *Lu. longipalpis* is a New World species and natural vector of *Leishmania infantum* parasites. The separation between primitive *Phlebotomus* and *Lutzomyia* occurred approximately 200 million years ago [43].

Previous studies have shown that host phylogeny is a strong driver of microbiota structure in the insect gut [27, 44, 45]. Recently, it has been suggested that the host genotype is the major modulator of the wild population of *Phlebotomus* sand fly gut microbiota [28]. In this context, we explored the role of host species in the microbiota diversity of *Leishmania* free Old and New World sand fly species reared under the same conditions. These results indicate that adult *Ph. papatasi* and *Ph. duboscqi* have a similar microbiome composition, which is distinct from that of adult *Lu. longipalpis*. Furthermore, we showed that *Ph. papatasi* and *Ph. duboscqi* are hosts for different bacterial genera. It has been demonstrated that microbiome composition is positively correlated with phylogenetic relatedness between hosts, that is, closely related host species tend to possess more bacterial clades in common than distantly related hosts [46]. These findings support our hypothesis that host species determine the composition of the prokaryotic microbiota in sand flies.

Our results show that *Proteobacteria* was the main phylum in the studied samples. These findings are consistent with the data from a recent meta-analysis of sand fly associated bacteria, which revealed that > 57% and 47% of the identified bacteria belonged to the *Proteobacteria* phylum in New and Old World sand fly species, respectively [47]. The distinct composition of the bacterial communities of *Ph. papatasi* and *Ph. duboscqi* was driven by two ASVs belonging to Rhizobiaceae and Yersiniaceae (genus *Serratia*). However, *Lu. longipalpis* was characterized by a specific bacterial structure dominated by *Achromobacter*, *Tsukamurella*, *Staphylococcus*, and *Stenotrophomonas*. Further studies are required to investigate the contributions of different bacterial taxa to the life traits of sand fly species, considering the contrasting evidence for the role of endosymbiotic bacteria in the infectivity and survival of *Leishmania* [14, 17].

A few bacteria from sand fly food grew slightly in some larvae and adults. The observed differences in microbiota communities between the gut and used food may be explained by insect behavioral adaptations, which further promote the dominant gut bacterial taxa, including coprophagy (eating of feces), trophallaxis (the transfer of food or other gut fluids through mouth-to-mouth or anus-to-mouth feeding), and maternal smearing of the bacterial gut on the eggshell, which is subsequently consumed by the offspring [48, 49]. Additionally, host sand flies can influence the physicochemical conditions of the gut, which may result in differential bacterial growth [50]. Based on our results, the modes of bacterial acquisition and growth appear to be similar among *Phlebotomus* species, which are distinct from *Lu. longipalpis*.

This experiment was repeated twice to accurately test our bacterial host phylogeny hypothesis, and a distinct composition of the microbiome among the same species was identified. The use of different batches of larval foods containing different bacterial contents may explain the present finding, even if few bacteria from the food grew slightly in some larvae and adults. The latter may interact with the host microorganisms and completely change the microbiota structure. Our findings are in agreement with those of previous studies suggesting a role for diet in gut bacterial composition in insects [26]. It has been demonstrated that the interspecific interactions among microorganisms, by which microbes compete for nutrients and space, may change the microbiota content associated with insects [51, 52]. Theoretical synthesis research has proposed the importance of host control (that is, partner choice and fidelity) in the maintenance of mutualistic associations [53, 54]. However, it is necessary to mention that the remarkable divergence in microbiota structure between the two biological replicates may also reveal a large individual variation in the bacterial composition of the studied sand fly species, and we further propose that the bacterial communities are only loosely and temporarily associated with these sand flies. Because we pooled samples in our experiment, more studies should examine the microbial communities of individual sand flies to assess changes between individuals.

Females of two sand fly genera, *Phlebotomus* and *Lutzomyia*, are of medical importance as the only established vectors of *Leishmania* species that are pathogenic to humans [55]. In the present study, bacterial communities in *Leishmania*-uninfected and -infected sand flies were identified and compared using Illumina MiSeq sequencing. The gut microbiome has previously been reported to either enhance or inhibit parasite activity depending on the species of bacteria and thus has the potential to alter vector competence [56]. The present study showed that *Serratia* genus decreased significantly and disappeared completely in infected *Ph. duboscqi* and *Ph. papatasi*, possibly favoring *L. major* development. Indeed, this bacterial genus has been demonstrated to negatively affect *L. infantum* and *Leishmania braziliensis* by inducing lysis of the parasite cell membrane and co-infected with *Lu. longipalpis* in vitro [57, 58]. The antagonistic interaction between bacteria and *Leishmania* parasites acted as a shaping force of the community assembly. Very recent work has focused on how resident microbiota can affect the *Leishmania* infection of the vector with surprising results. Basically, it was found that the sand fly microbiota is fundamental for *Leishmania* development and transmission. One paper suggests that the removal of the microbiota alters the osmolarity of the intestinal environment and is thus deleterious for the *Leishmania* development [59]. Since we do not know exactly which mechanisms are responsible for this interaction/dependence between *Leishmania* and microbiota in vitro cultures and in vivo, this may be considered an open field of research.

Our results show that the microbial charge was markedly reduced after adult emergence and increased significantly after blood feeding. These findings are in line with previous data indicating that the microbial charge immediately and markedly decreases after adult emergence [14, 15] and increases after blood feeding [15, 17]. Additionally, the difference in bacterial load between uninfected and infected sand flies suggests a quantitative interaction with *Leishmania* parasites. It has already been demonstrated that any manipulation that reduces the size/diversity of natural microbiota affects the ability of *Leishmania* to establish infections in sand flies [17, 60]. However, studies investigating the bacterial load separately did not find any significant impact on infection rates [34].

## Conclusions

This is the first report on the gut bacterial microbiomes of *Phlebotomus* and *Lutzomyia* sand flies reared under the same conditions for many generations. Our analysis showed that adult *Ph. papatasi* and *Ph. duboscqi* have a similar microbiome composition, which is distinct from that of adult *Lu. longipalpis*, indicating the role of phylogeny in the composition of insect gut microbiota. The experiment was repeated twice, and the same host phylogeny conclusions were obtained even when a distinct composition of the microbiome among the same species was identified. However, differences in the bacterial diversity and abundance of gut bacterial communities have been reported between uninfected and infected sand flies. Based on these results, future studies should focus on the role of these microorganisms in the biology of sand fly species, considering the contrasting evidence for the role of the detected bacteria in the infectivity and survival of *Leishmania*.

### Supplementary Information

**Additional file 1: Figure S1.** **a**. PCoA plot illustrating beta diversity distance matrices of the Bray-Curtis distance comparing the sample distribution among the three species, experiment 1. **b**. PCoA plot illustrating beta diversity distance matrices of the Bray-Curtis distance comparing the sample distribution among the three species, experiment 2.

**Additional file 2: Figure S2.** **a**. The relative bacterial quantities of three laboratory-reared sand flies, experiment 1. **b**. The relative bacterial quantities of three laboratory-reared sand flies, experiment 2.

**Additional file 3: Figure S3.** The relative bacterial quantities of uninfected and infected three laboratory-reared sand flies

## Data Availability

The microbiome sequences data in this study were registered in NCBI/GenBank/DDBJ database at SAMN35318066–SAMN35318107.
