# Supplementary figures and images for: Effects of host species on microbiota composition in Phlebotomus and Lutzomyia sand flies
Source: Parasit Vectors. 2023 Aug 31;16:310. doi: 10.1186/s13071-023-05939-2 (PMC10472604; doi:10.1186/s13071-023-05939-2)

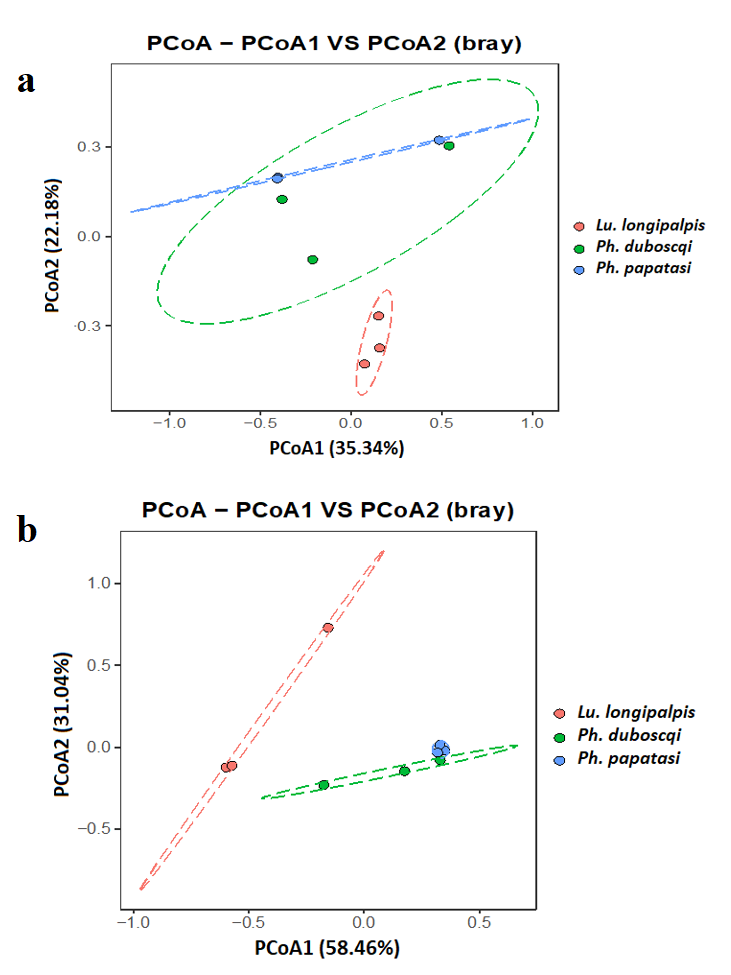

Supplement: Supplementary file 1 — Additional file 1: Figure S1. a. PCoA plot illustrating beta diversity distance matrices of the Bray-Curtis distance comparing the sample distribution among the three species, experiment 1. b. PCoA plot illustrating beta diversity distance matrices of the Bray-Curtis distance comparing the sample distribution among the three species, experiment 2. [file 13071_2023_5939_MOESM1_ESM.tif]

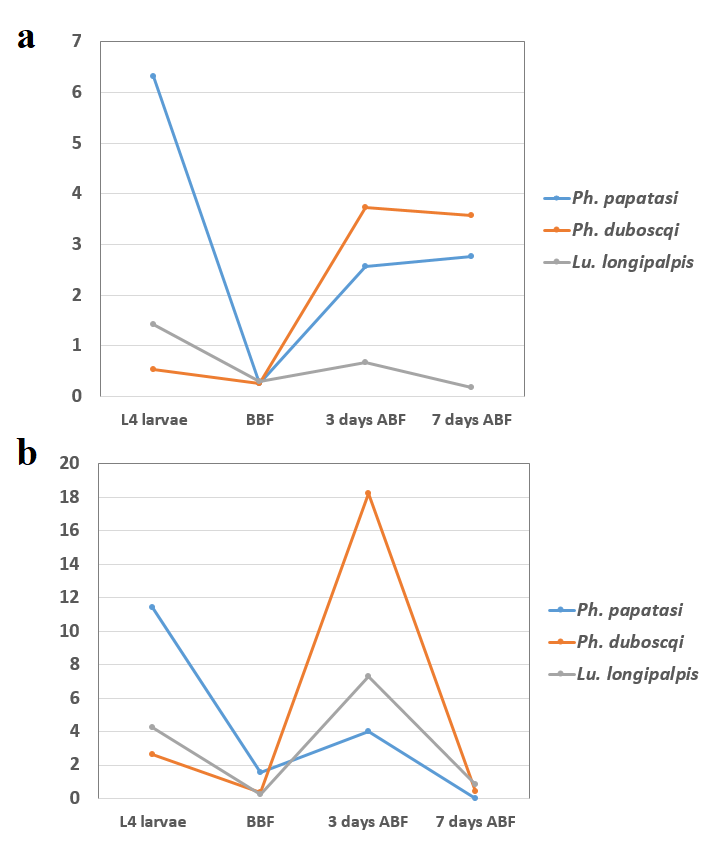

Supplement: Supplementary file 2 — Additional file 2: Figure S2. a. The relative bacterial quantities of three laboratory-reared sand flies, experiment 1. b. The relative bacterial quantities of three laboratory-reared sand flies, experiment 2. [file 13071_2023_5939_MOESM2_ESM.tif]

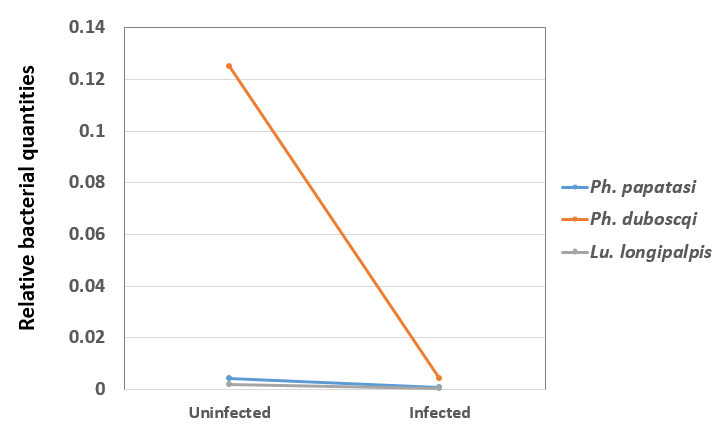

Supplement: Supplementary file 3 — Additional file 3: Figure S3. The relative bacterial quantities of uninfected and infected three laboratory-reared sand flies [file 13071_2023_5939_MOESM3_ESM.tif]
